# Supplementary material for: Contrasting functions of ATP hydrolysis by MDA5 and LGP2 in viral RNA sensing
Source: J Biol Chem. 2024 Feb 1;300(3):105711. doi: 10.1016/j.jbc.2024.105711 (PMC10909783; doi:10.1016/j.jbc.2024.105711)
Supplement: MDA5_LGP2 ATPase_SI_JBCrev [file mmc2.pdf]

SUPPORTING INFORMATION for

Contrasting functions of ATP hydrolysis by MDA5 and LGP2 in viral RNA sensing

Rahul Singh, Yuan Wu, Alba Herrero del Valle, Kendra E. Leigh, Sai Mong, Mark T. K. Cheng, Brian J. Ferguson, Yorgo Modis

This PDF files includes:

Figure S1

Figure S2

Figure S3

Figure S4

Other Supporting Information for this manuscript:

Source data file for Figures 1-4, Figure 6, and Figures S1-S4.

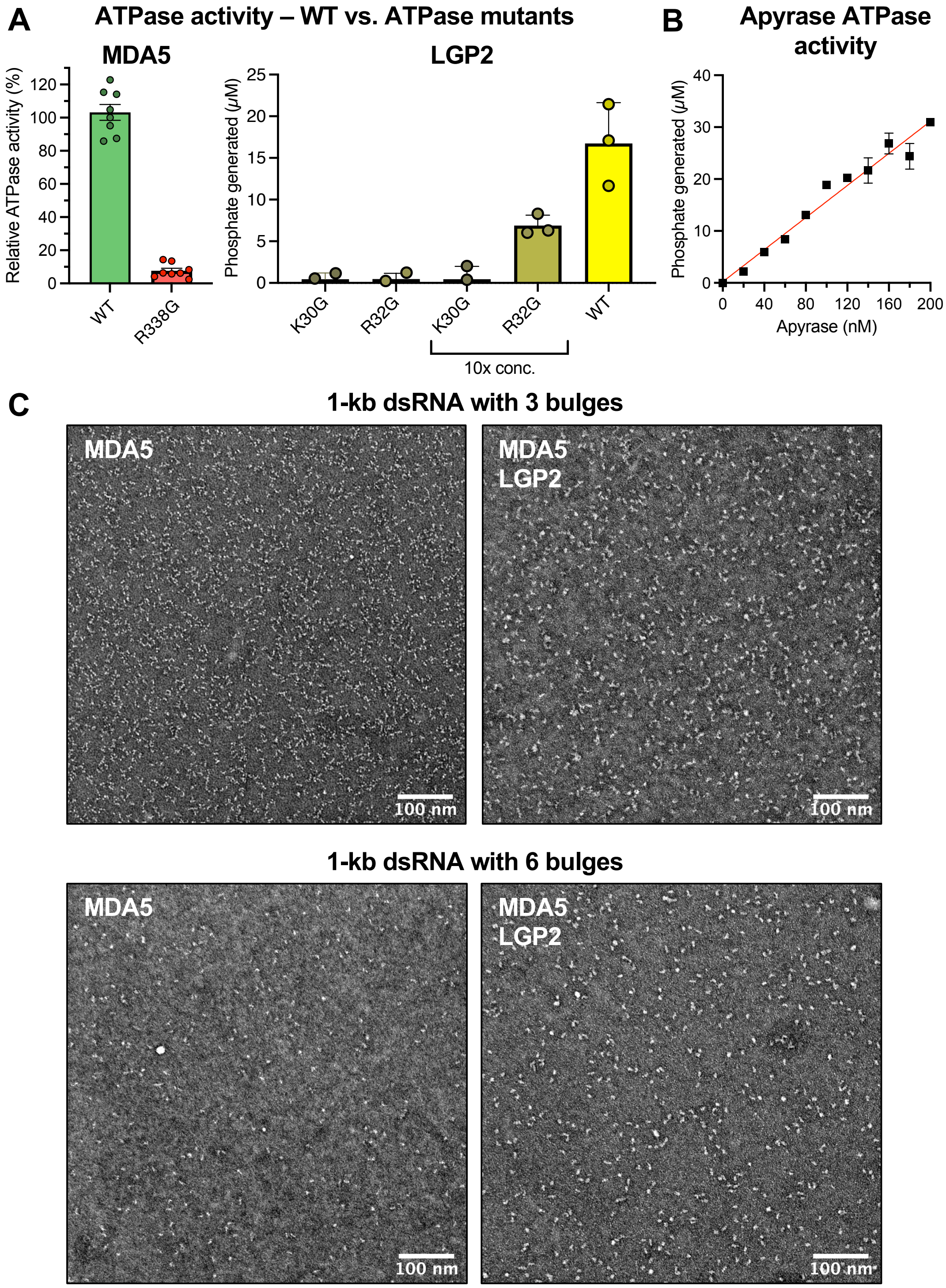

Figure S1. ATPase assays of MDA5 and LGP2 and electron micrographs with dsRNA containing bulges. (**A**) ATPase activities of R338G MDA5 (left), and K30G LGP2 and R32G LGP2 (right), in the presence of 1-kb dsRNA. Error bars show the s.e.m. of eight and three independent experiments for MDA5 and LGP2, respectively. (**B**) ATPase activity of apyrase, a monomeric ATPase, as a function of apyrase concentration. (**C**) Negative stain electron micrographs of filaments formed in the presence of 73 nM MDA5, 73 nM LGP2, 1 mM ATP-$MgCl_2$, and 4.4 nM 1-kb dsRNA with 3 or 6 single-nucleotide bulges. Scale bars, 100 nm.

A **Bio-layer interferometry (BLI) with 300-bp dsRNA**

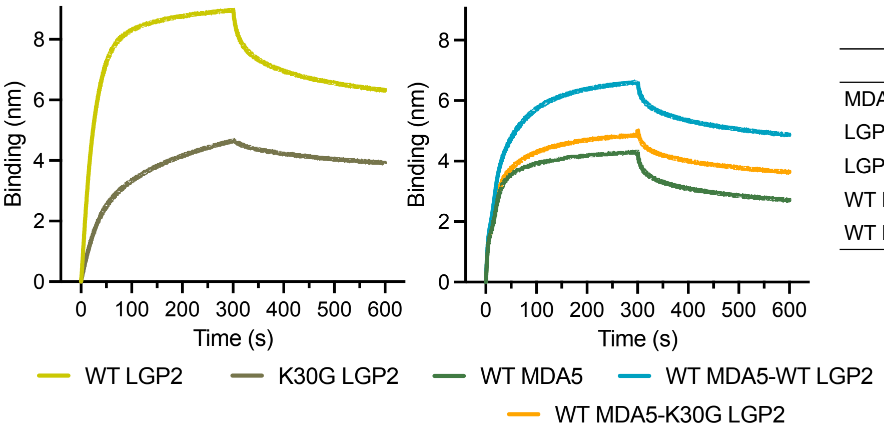

300 bp dsRNA	
	K_d (nM)
MDA5	1310
LGP2	93.8
LGP2 K30G	1150
WT MDA5 + WT LGP2	495
WT MDA5 + LGP2 K30G	1050

BLI with 40-nt ssRNA

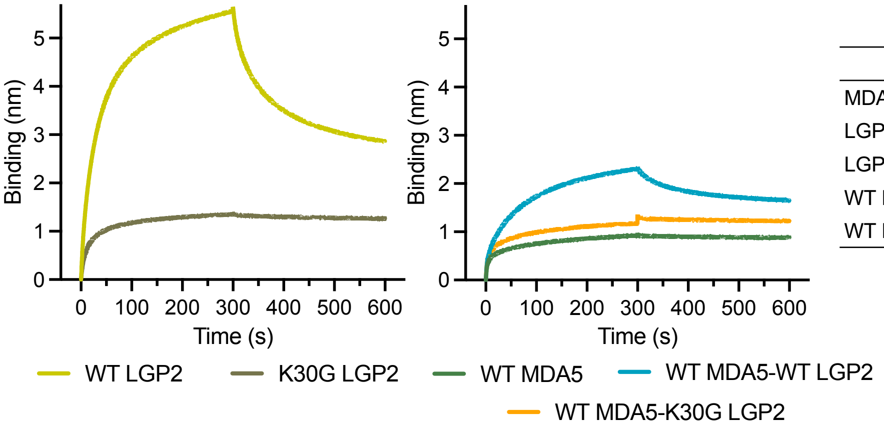

40 nt ssRNA	
	K_d (nM)
MDA5	2600
LGP2	402
LGP2 K30G	1400
WT MDA5 + WT LGP2	408
WT MDA5 + LGP2 K30G	1840

B **BLI with 1 kb dsRNA and LGP2 with mutations at predicted MDA5 interface**

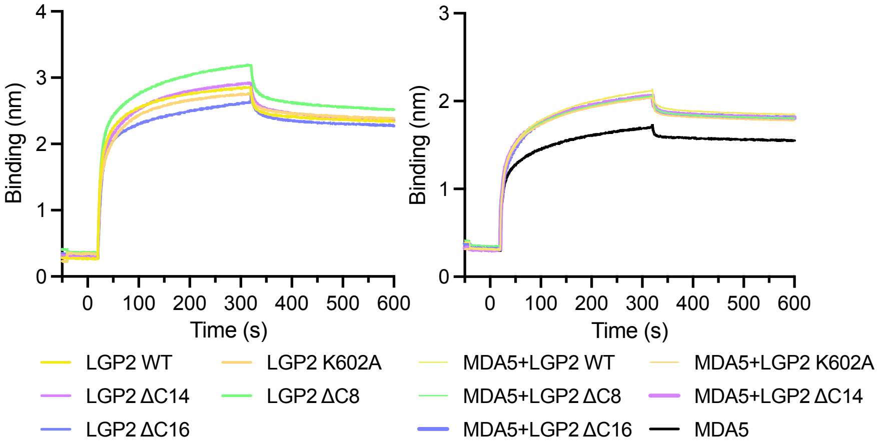

1 kb dsRNA	
	K_d (nM)
LGP2	48.0
LGP2 K602A	64.3
LGP2 ΔC8	40.1
LGP2 ΔC14	71.6
LGP2 ΔC16	95.3
MDA5	1866
MDA5+LGP2	454
MDA5+LGP2 K602A	535
MDA5+LGP2 ΔC8	478
MDA5+LGP2 ΔC14	322
MDA5+LGP2 ΔC16	563

Figure S2. RNA binding assays with MDA5 and different LGP2 variants and RNA ligands. (**A**) Bio-layer interferometry with 3'-biotinylated 300-bp dsRNA (top) or 40-nt ssRNA (bottom) immobilized on a streptavidin biosensor and MDA5, LGP2 or MDA5-LGP2 mixtures in the mobile phase. Dissociation constants derived from the curves are tabulated. (**B**) Bio-layer interferometry with 3'-biotinylated 1-kb dsRNA immobilized on a streptavidin biosensor and MDA5, LGP2 or MDA5-LGP2 mixtures in the mobile phase. Dissociation constants derived from the curves are tabulated.

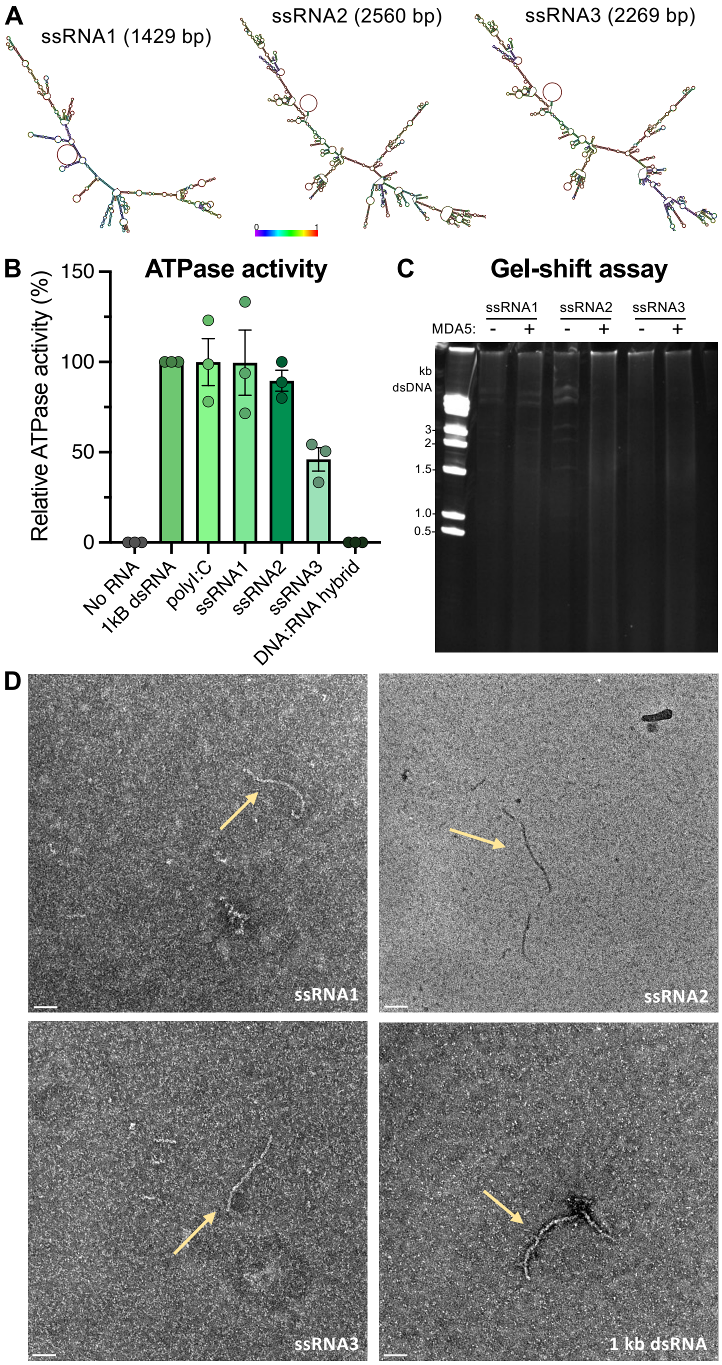

Figure S3. ATPase and RNA binding assays for MDA5 and long ssRNAs with complex secondary structure. (**A**) Synthetic ssRNAs designed to form complex, highly base-paired secondary structures. Minimum Free energy secondary structures predicted by RNAfold [http://rna.tbi.univie.ac.at] are shown, colored by base pairing probability. See source data file in Supporting Information for sequences. (**B**) MDA5 ATPase activity in the presence of ssRNA1-3, along with high-molecular weight poly(**I**:C) and a 1-kb DNA:RNA hybrid, expressed as percent of ATPase activity with 1-kb dsRNA. Error bars show the s.e.m. of three independent experiments. (**C**) Polyacrylamide gel-shift assay for binding of MDA5 to ssRNA1-3. (**D**) Negative-stain electron micrographs of MDA5 filaments formed in the presence of ssRNA1-3. Scale bars, 50 nm.

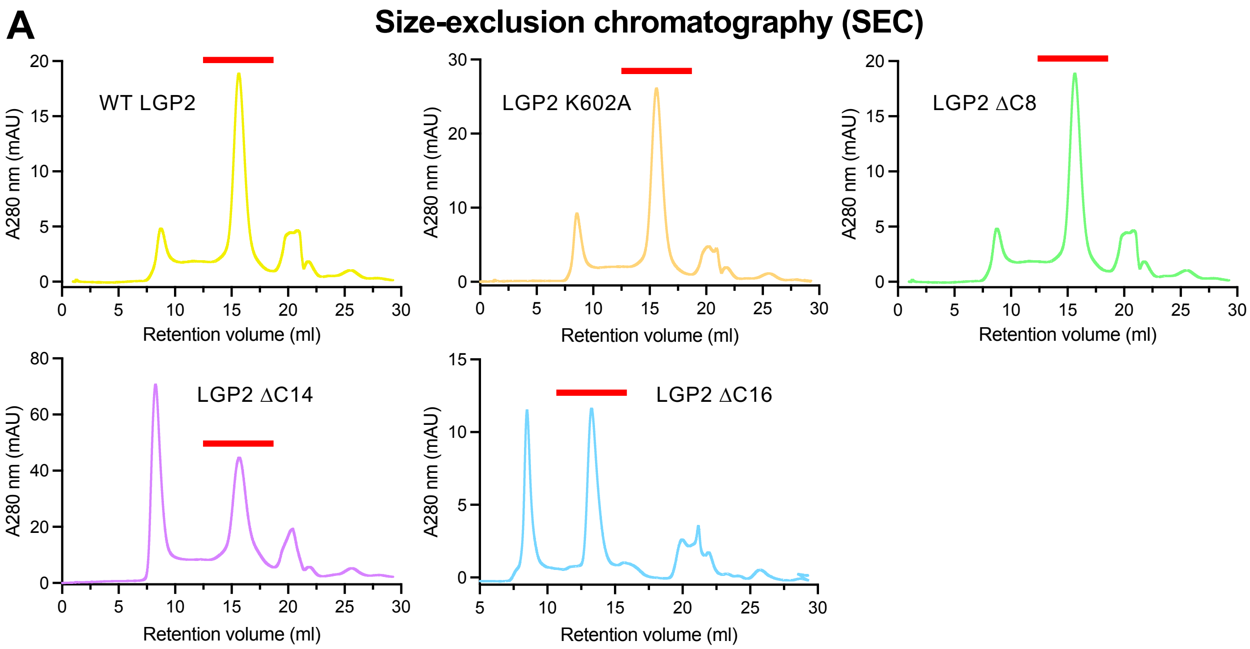

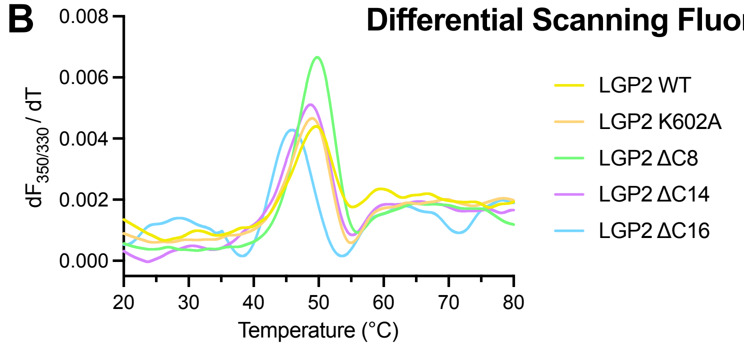

Melting Temperature (T_m)

	T_m (˚C)
LGP2	49.5
LGP2 K602A	49.3
LGP2 ΔC8	49.7
LGP2 ΔC14	48.7
LGP2 ΔC16	45.6

Figure S4. Size-exclusion chromatography (SEC) and differential scanning fluorimetry (DSF) or LGP2 C-terminal deletion and capping loop mutant. (**A**) SEC traces of wild-type, K602A, ΔC8, ΔC14, and ΔC18 LGP2. Red lines indicate the elution volume ranges from which LGP2 fractions were collected for biochemical assays. (**B**) DSF of 25 μM LGP2 (wild-type, K602A, ΔC8, ΔC14, or ΔC16). Intrinsic protein fluorescence at 330 nm and 350 nm was measured and the fluorescence ratio plotted as a function of temperature. The temperature value at the peak of the first derivative of the fluorescence ratio versus temperature function was taken as the melting temperature (T_m).
